# All-fiber ultrafast amplifier at 1.9 μm based on thulium-doped normal dispersion fiber and LMA fiber compressor

**DOI:** 10.1038/s41598-021-02934-4

**Published:** 2021-12-08

**Authors:** Vasilii Voropaev, Daniil Batov, Andrey Voronets, Dmitrii Vlasov, Rana Jafari, Aleksandr Donodin, Mikhail Tarabrin, Rick Trebino, Vladimir Lazarev

**Affiliations:** 1 Science and Education Center for Photonics and IR-Technology, Bauman Moscow State Technical University, Moscow, Russia 105005; 2 Georgia Institute of Technology, Atlanta, GA 30332 USA; 3 Aston Institute of Photonic Technologies, Aston University, Birmingham, B4 7ET UK; 4 Frequency Standards Laboratory, P. N. Lebedev Physical Institute of the Russian Academy of Sciences, Moscow, Russia 119991

**Keywords:** Mode-locked lasers, Ultrafast lasers, Fibre lasers

## Abstract

The duration reduction and the peak power increase of ultrashort pulses generated by all-fiber sources at a wavelength of $1.9\,\upmu \hbox {m}$ are urgent tasks. Finding an effective and easy way to improve these characteristics of ultrafast lasers can allow a broad implementation of wideband coherent supercontinuum sources in the mid-IR range required for various applications. As an alternative approach to sub-100 fs pulse generation, we present an ultrafast all-fiber amplifier based on a normal-dispersion germanosilicate thulium-doped active fiber and a large-mode-area silica-fiber compressor. The output pulses have the following characteristics: the central wavelength of $1.9\,\upmu \hbox {m}$, the repetition rate of 23.8 MHz, the energy per pulse period of 25 nJ, the average power of 600 mW, and a random output polarization. The pulse intensity and phase profiles were measured via the second-harmonic-generation frequency-resolved optical gating technique for a linearly polarized pulse. The linearly polarized pulse has a duration of 71 fs and a peak power of 128.7 kW. The maximum estimated peak power for all polarizations is 220 kW. The dynamics of ultrashort-pulse propagation in the amplifier were analyzed using numerical simulations.

## Introduction

Ultrafast thulium-doped fiber-laser sources at the wavelength of $1.9\,\upmu \hbox {m}$ have attracted great interest due to their wide range of potential applications^1^, including remote sensing, precision frequency-domain spectroscopy^2^, and breath analysis^3^. Such systems are compact, reliable, easy to align, and environmentally stable when using polarization-maintaining fibers. Most of the aforementioned applications require a coherent supercontinuum in the mid-IR region that is conveniently achieved using ultrafast Tm-doped fiber lasers^4,5^. For the generation of broadband coherent supercontinua, nonlinear media with anomalous group-velocity dispersion (GVD) are desirable^4,6^. However, the product of the energy and duration of the pulses should not exceed a certain value, otherwise the process of modulation instability precedes a strong broadening of the spectrum and degrades its temporal coherence^6,7^. Thus, one of the key factors for the bandwidth increase of the coherent supercontinuum achieved in nonlinear media with anomalous GVD is the pulse duration decrease.

**Figure 1:**
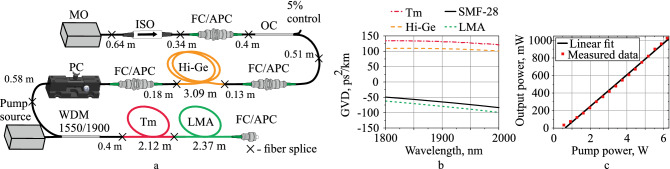
(**a**) Schematic of the thulium-doped all-fiber amplifier. All the unspecified segments of fibers are SMF-28 fibers. *MO* master oscillator, *ISO* isolator, *OC* coupler, *Hi-Ge* fiber with a high concentration of germanium oxide, *PC* polarization controller, *WDM* wavelength-division multiplexer, *Tm* thulium-doped germanosilicate fiber, *LMA* large-mode-area silica fiber; (**b**) Group velocity dispersion (GVD) as a function of wavelength for the SMF-28 (black curve), Tm-doped fiber (red dash-dot curve), Hi-Ge (orange dash curve) and LMA (green dot curve); (**c**) The dependence of output power on pump power where red dots are measured data and the black curve is a linear fit.

Recently, significant progress has occurred in the development of ultrafast fiber-laser systems at $1.9\,\upmu \hbox {m}$ with pulse durations less than 150 fs and peak powers higher than 10 kW^4,8–11^ and commonly used to generate broadband coherent supercontinua. In thulium-doped fiber-laser systems, various techniques and their combinations are used to achieve such pulse characteristics, including the use of large-mode-area (LMA) active fibers^8^, nonlinear pulse compression^9–11^, the chirped-pulse amplification technique^4,10,11^, etc. In some works, pulses with such parameters are achieved without using an amplifier^12,13^. Extremely high values of peak power (MW-GW level) can be obtained by using fibers with very large mode areas (large-pitch fibers^14^) and nonlinear compression in gas-filled hollow-core fibers^15,16^. In the supplementary materials (Fig. 1) we have given a comparison of the pulse characteristics of the mentioned works together with the design features. Another well-known approach for obtaining such pulse characteristics is to amplify pulses in fibers with normal GVD, leading to a significant spectral and temporal broadening of a pulse while maintaining the pulse uniformity^17^. Then pulses are compressed in fibers with anomalous GVD to achieve low duration and high peak power characteristics. However, very few fiber laser systems based on thulium-doped normal dispersion fibers have been experimentally embodied^18,19^. These setups feature durations of more than 600 fs, which are less promising for coherent supercontinuum generation.

In this work, for the first time to the best of our knowledge, we present a system for ultrashort pulse amplification and compression based on the combination of an active germanosilicate thulium-doped fiber with a normal GVD and an LMA fiber with an anomalous GVD. Using numerical simulations, the dynamics of pulses inside the amplifier are analyzed and the use of a pulse stretcher before the amplifier is justified. The use of a germanosilicate thulium-doped fiber with normal GVD as an active medium significantly expands the pulse spectrum during the amplification process. This broadening helps to achieve a shorter pulse duration than that fundamentally achievable at the input of the amplifier. The pulse undergoes compression in the LMA fiber, resulting in a group of pulses with a minimal main-pulse duration of 71 fs. An LMA fiber has a lower nonlinear coefficient compared to standard single-mode fiber, which helps to reduce nonlinear distortion of the pulse and to maintain the high peak power of the achieved pulse (220 kW). The pulse intensity-and-phase profile was measured via the second-harmonic-generation (SHG) frequency-resolved optical gating (FROG)^20^.

## Experimental setup

Figure 1a shows the schematic of the amplifier with the lengths of all relevant fibers. The GVD as a function of wavelength for all fibers (Fig. 1b), except the LMA, was measured by the method described in^21^. The dependence of the amplifier output power on the pump power is shown in Fig. 1c. The maximum output power was approximately 1 W at 6 W pump power.

The master oscillator (MO) was the stretched-pulse Tm-doped all-fiber ring laser with hybrid mode-locking, a detailed description of which is given in the work^22^. The nonlinear polarization evolution and single-wall carbon nanotubes were used as the mode-locking mechanisms. The average power of the MO in the mode-locking regime was 6 mW. The pulse repetition rate was 23.8 MHz. The spectrum and pulse autocorrelation trace from the MO were measured after the FC/APC connector after the isolator (See Fig. 1a) and are shown in the section of “Experimental results”. The maximum intensity wavelength was 1899.5 nm, the spectral full width at half maximum (FWHM) was 21.66 nm. The autocorrelation trace had a Gaussian shape with FWHM of 465 fs, which corresponds to a 328.8 fs FWHM of the pulse.

A polarization-independent isolator (ISO) was used to prevent back reflections to the MO cavity. FC/APC optical adapters were used for the connection between different parts of the amplifier. The coupler (95/5) directed 5% of the radiation to the photodetector for controlling the generation regime and had a 5% of losses. The 3.09 m-long-fiber with a high concentration of germanium ($\Delta \hbox {n} \approx 0.0324$, 30 wt% germanium oxide in the core, core diameter is $2.2\,\upmu \hbox {m}$) (Hi-Ge) with the GVD value of $108\,\hbox {ps}^{2}/\hbox {km}$ at $1.9\,\upmu \hbox {m}$ was used to obtain stretched pulses with a positive chirp before amplification. According to the simulation, the use of this fiber allows an increase in the peak power and reduces the pulse pedestal. A theoretical comparison with the case without this fiber is given in the “Simulation results” section. Hi-Ge-SMF splicing losses are about 10%. The mechanical polarization controller was placed after the Hi-Ge fiber because the propagation process strongly depends on the radiation polarization state, presumably due to the dependence of the nonlinear refractive index on the polarization state of the radiation^23^.

The CW pump source at a wavelength of 1550 nm (Erbium-Ytterbium-doped fiber amplifier of the laser diode) was connected to the wavelength-division multiplexer (WDM) to inject pumping into the active fiber. The amplifier was based on a normal dispersion step-index ($\Delta \hbox {n}\approx 0.045$, core diameter is $2.2\,\upmu \hbox {m}$) $\hbox {Tm}^{3+}$-doped germanosilicate (0.9 wt% thulium, 36 wt% $\hbox {GeO}_{2}$) fiber with a normal GVD of $130.55\,\hbox {ps}^{2}/\hbox {km}$ at 1900 nm. The length of the $\hbox {Tm}^{3+}$-doped fiber was 2.12 m and chosen so that $\approx 98\%$ of the pump power was absorbed.

The LMA pigtail ($\Delta \hbox {n} \approx 0.0022$, core diameter of $20\,\upmu \hbox {m}$) was used to compress the pulses. The calculated GVD of the LMA fiber is $-79.58\,\hbox {ps}^{2}/\hbox {km}$ at 1900 nm. The LMA fiber is a PANDA-type fiber, but it did not work in the polarization-maintaining regime in our experiments due to random polarization at the fiber input. This type was not chosen on purpose, but due to the availability of only such a fiber. The splice between the LMA and the thulium-doped fibers introduces losses due to the difference in mode-field diameters (20.6 and $5.06\,\upmu \hbox {m}$), experimentally estimated to be 30%. The length of the LMA fiber (2.37 m) was selected experimentally to achieve the shortest pulse duration with 600 mW average output power. The justification of the average output power will be explained based on the simulation results. All used fibers, except SMF-28, were manufactured at Dianov Fiber Optics Research Center and Devyatykh Institute of Chemistry of High-Purity Substances of the Russian Academy of Sciences.

## Simulation results

**Figure 2:**
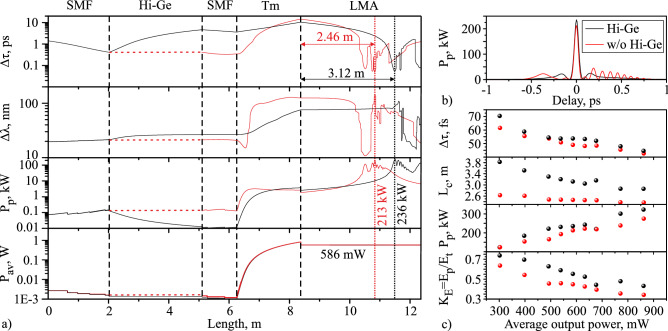
(**a**) Simulated evolution of temporal ($\Delta \uptau$) and spectral ($\Delta \uplambda$) FWHM, peak ($\hbox {P}_{\mathrm{p}}$) and average ($\hbox {P}_{{\mathrm{av}}}$) power of the pulse or pulse group (if several pulses are formed) in the amplifier with Hi-Ge (black curve) and without Hi-Ge fiber (red curve); Calculated pulse intensity profiles (**b**) for case with Hi-Ge fiber and without it with the average output power of 586 mW at the compression point; (**c**) Simulated the pulse durations ($\Delta \uptau$), comparison of compression lengths ($\hbox {L}_{\mathrm{c}}$), peak powers ($\hbox {P}_{{\mathrm{p}}}$), and the ratios of the energy contained in the main pulse to the total energy per pulse period ($\hbox {K}_{\mathrm{E}}=\hbox {E}_{\mathrm{p}}/\hbox {E}_{\mathrm{t}}$) for cases with Hi-Ge fiber (black dots) and without Hi-Ge fiber (red dots) at compression points at different average output powers.

Using the numerical model described in the “Methods” section, we calculated the dynamics of the pulse parameters in the amplifier. These parameters are temporal and spectral FWHM, peak and average powers of the pulse or pulse group (if several pulses are formed) propagating in the amplifier (Fig.2a) with and without Hi-Ge fiber with 586 mW output average power.

The first section of the amplifier consists of an SMF-28 fiber of passive components (isolator, coupler), where the pulse compresses in the time domain, the spectral FWHM increases slightly, the peak power increases, and the average power decreases due to the loss of components. The pulse undergoes a slight spectral broadening from 21.3 to 26.5 nm while propagating in Hi-Ge fiber, the pulse duration increases from 412 fs to 4.66 ps, and the peak power decreases from 127 to 12 W. The average output power at the Hi-Ge output is reduced to 1.1 mW, mainly due to losses in the splices and connectors. The measurement of the pulse duration experimentally at this point is difficult due to the product of the peak power and the average power ($0.0132\,\hbox {W}^2$) being less than the sensitivity threshold of the used autocorrelator ($1\,\hbox {W}^2$). In the SMF section of WDM before the active fiber, the pulse duration in the case with Hi-Ge fiber decreases to 3.6 ps, and in the case without Hi-Ge fiber, the pulse duration decreases from 412 to 350 fs, while the spectral FWHM and peak power are almost unchanged in both cases.

In the active fiber in the case with the Hi-Ge fiber, the pulse duration increases to 10.4 ps, and at the same time, due to the action of self-phase modulation the spectrum is broadened to 75 nm. In the case without a Hi-Ge fiber at the initial stage of propagation, the pulse duration slightly decreases with narrowing of the spectrum. Then there is an increase in the pulse duration and the spectral FWHM. At the output of the active fiber, the FWHM of the spectrum is 126 nm, and the pulse duration is 14.45 ps. In both cases, the average power at the end of the active fiber is $\approx 840\,\hbox {mW}$.

**Figure 3:**
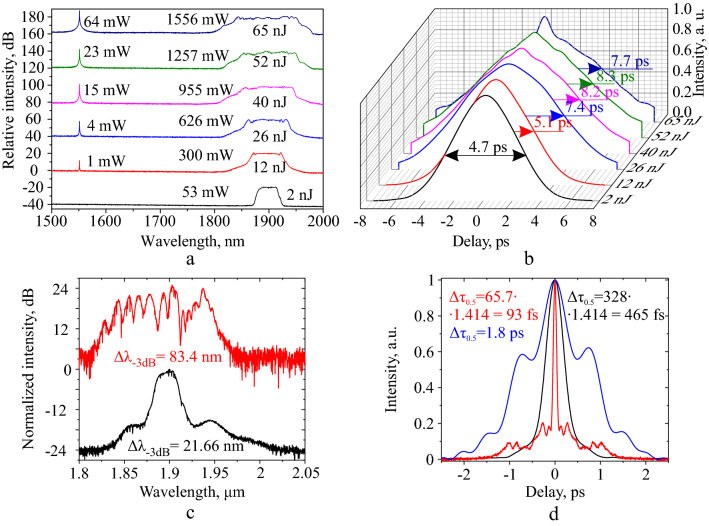
Spectra (**a**) and autocorrelation traces (**b**) measured at the output of the active fiber at different pump powers; Spectra (**c**) and intensity autocorrelation traces of the pulses (**d**); Black curves—from master-oscillator; Red and Blue curves—from the amplifier at 600 mW output power. Blue curve on the graph (**b**)—autocorrelation trace of long pulse from amplifier achieved by polarization controller (PC) adjustment.

**Figure 4:**
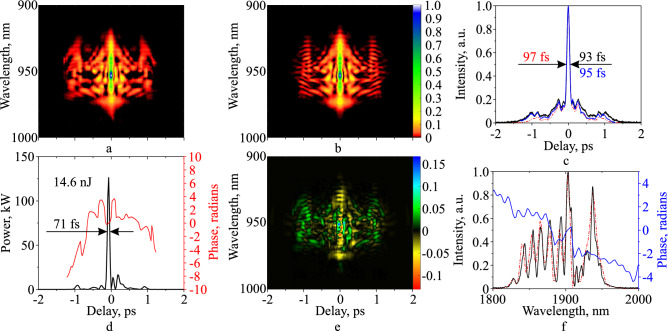
Characteristics of the shortest horizontal polarized pulses with the power of 350 mW at the amplifier output at an average output power of 600 mW for all polarization states. Measured FROG trace (**a**), retrieved FROG trace (**b**), retrieved pulse intensity versus time (**d**, black), retrieved pulse phase versus time (**d**, red), the difference between measured and retrieved traces (**e**), retrieved autocorrelation (**c**, red dash), measured autocorrelation trace (**c**, black) and autocorrelation trace obtained by integrating of measured FROG trace (**c**, blue), retrieved spectrum (**f**, red dash), spectral phase (**f**, blue) and the spectrum measured by OSA (**f**, black). The x-axes in figures (**a**) and (**b**) are the same as in (**c**) and (**d**).

During pulse compression, a group of pulses is formed in the LMA fiber due to the action of nonlinear effects and higher-order dispersion. We determine the spectral width and duration of the pulse group by the full width at half of the maximum amplitude of the spectrum and intensity. Near the compression point, the dependence of the pulse group duration and spectral width has rapid oscillations and, in some places, discontinuous changes. This behavior is associated with a change in the amplitude of pulses in the group, when the amplitude of one of the pulses becomes slightly bigger than the half amplitude of the main pulse, there is a sharp increase in the duration of the pulse group. More detailed illustrations of the pulse compression dynamics in the two amplifier schematics are given in the supplementary materials (Figs. 2–4). The pulse duration in LMA fiber has several minima in both cases. The point with the minimum pulse duration is taken as the compression point in this paper. So, the compression length in the case without a pulse stretcher is 2.46 m, and in the case using a pulse stretcher is 3.12 m. The pulse duration at the compression point in the case without a pulse stretcher is 49 fs, and in the case using a pulse stretcher it is 53.8 fs. A comparison of the pulse intensity profile at the compression point for the two cases is shown in Fig. 2b at an output power of 586 mW. As a result of compression in the LMA fiber, a group of pulses is formed, consisting of the main pulse and several pulses with lower amplitude. The pulse peak power at the compression point is 236 kW with the stretcher and 213 kW without one.

Figure 2c shows a comparison of the following parameters: compression lengths, the pulse durations, peak powers, and the ratios of the energy contained in the main pulse to the total energy per pulse period ($\hbox {K}_{\mathrm{E}}$) for two cases (with and without Hi-Ge) at compression points for different output radiation powers. As the output power increases, the main pulse duration and compression length decrease, and the peak power of the main pulse increases. Also, with an increase in the average output power, the ratio of the main pulse energy to the total energy decreases. Taking into account that the peak power of the pulse and the $\hbox {K}_{{\mathrm{E}}}$ according to the calculations are higher in the case with Hi-Ge fiber, we decided to carry out all experiments using this fiber. Moreover, according to simulations, with 600 mW output average power, about 55% of the radiation energy forms the main pulse with 236 kW peak power. Thus, we decided to experimentally investigate the compression point at an output power of about 600 mW.

## Experimental results

First, we measured the characteristics immediately after the active fiber. As the pulse propagates in the active fiber, its duration increases, and the spectrum significantly broadens^24^. Figures 3a, b show the measured broadening of the spectrum and an increase in the pulse duration at the output of the active fiber with an increase in the pump power. Thus, at a pump power of $\approx 4\,\hbox {W}$ at the output of the active fiber, the spectrum had FWHM of 92 nm and average power was 955 mW, and at a pump power of $\approx 7\,\hbox {W}$, spectral FWHM was 114 nm and average power was 1.55 W^24^. The duration of the autocorrelation was 8.2 ps with an output power of about 955 mW, which is less than that obtained in the simulation.

Next, we spliced the LMA fiber and experimentally found the compression length at an output power of 600 mW, which was 2.37 m. Compression length differs from the calculated one by 30% (3.12 m). The measured amplifier characteristics with an output power of 600 mW are shown in Fig. 3c, d in comparison with the characteristics of the master oscillator (black curves). The spectrum and autocorrelation trace of the pulse from the amplifier (Fig. 3a, b, red and blue curves) were measured at the end of the LMA fiber. At various settings of the PC (See Fig. 1a), autocorrelation traces of the output pulses from the amplifier had FWHM in the range from 93 fs (Fig. 3d, red curve) to 1800 fs (Fig. 3d, blue curve). The pulse changed at the various PC settings, probably due to the difference between nonlinear refractive indices ($\hbox {n}_2$) of fibers for the various polarization states (see “Discussion” section)^23^. The shortest pulses had a maximum intensity wavelength of 1902.7 nm, with a spectral FWHM of 83.4 nm (Fig. 3c, red curve). The minimum autocorrelation FWHM was 93 fs which corresponds to 65.7 fs pulse FWHM (assuming a Gaussian shape). However, the actual shortest pulse had a much more complex shape than a Gaussian, so it was necessary to measure the true temporal intensity profile using FROG to estimate the peak power and clarify the nature of the pulse pedestal of the autocorrelation trace associated with pre-pulses and post-pulses.

We measured the SHG FROG trace together with the autocorrelation and spectrum for horizontally polarized radiation with 350 mW average power. The vertical polarization state had an average power of 250 mW. To measure the FROG trace, we used a non-collinear geometry setup based on SHG in a BBO crystal with a thickness of 0.6 mm. Figure 4 shows the FROG measurement of the compressed pulses with the shortest duration at an average output power of 600 mW. The measured FROG trace (Fig. 4a) has the following parameters: $2048 \times 175$ points, delay resolution 1.27 fs, wavelength resolution 0.4 nm, delay range 2.6 ps, wavelength range 70 nm, the center wavelength 950 nm. The retrieved FROG trace (Fig. 4b) has $256 \times 256$ points. The delay axis extends in the range from $-2916.4$ to 2893.6 fs. The frequency axis is in the range from 293.4 to 337.12 THz, which corresponds to a wavelength range from 889.9 to 1022 nm.

The retrieved intensity pulse profile and corresponding phase are shown in Fig. 4d. Satellite pulses can be seen near the main pulse, associated with the excess accumulated nonlinearity by the pulse in the amplifier and also probably with the initially existing subpulses, which appear as small wings in the input autocorrelation and discussed in our previous work^22^. The center peak has a 71 fs duration and contains 64.5% (9.4 nJ) of the energy per pulse period (14.6 nJ) for the horizontal polarization state, and the corresponding pulse peak power is 128.7 kW. If we assume that the vertical polarization of the pulse has the same temporal intensity profile as the horizontal polarization of the pulse, then the peak power of the pulse with all polarizations can be estimated as 220 kW. The temporal phase has a clear quadratic component, indicating that additional compression to a shorter pulse could be possible, although much of this curve occurs for low-intensity satellites, which do not contribute to the FWHM pulse length.

Figure 4e shows the difference between measured and retrieved FROG traces. There is more structure in the retrieved trace than the measured trace. This indicates slight shot-to-shot pulse variations in the pulse intensity and/or phase vs. time, which wash out structure in measured FROG traces^25,26^. Due to the many more points in the FROG trace than the pulse, the FROG algorithm is able to see through the smearing. It accomplishes this by averaging over many different pulses. The result is that it yields a typical pulse in the train. Other pulses in the train and their corresponding FROG traces will have a similar structure but displaced slightly in time and/or frequency.

To relate the FROG measurements to an older, less detailed, but more familiar, measure, we compare autocorrelation traces (Fig. 4c) of the pulses. The FROG-measured autocorrelation trace, obtained by integrating the measured FROG trace with respect to wavelength, and retrieved autocorrelation traces show a good agreement in shape. The autocorrelation duration mismatch is 4.3%, the Pearson correlation coefficient between the two autocorrelation traces is 0.98. Slight differences between the mathematically equivalent measured autocorrelation trace and the autocorrelation trace obtained by integrating the measured FROG trace over all frequencies are due to random noise. Differences between the FROG-retrieved pulse’s autocorrelation and the other two autocorrelation traces are partly due to the rough nature of the autocorrelation as a measure of a pulse. They are also partly due to FROG’s ability to yield a typical pulse, rather than an average pulse (to understand this, note that the average spectral phase yields only the coherent artifact and so is highly undesirable). For a more detailed discussion of this interesting issue, which is beyond the scope of this publication, we refer the reader to Ref.^27^.

Finally, we also compare the FROG-retrieved spectrum with that measured directly by a Fourier-transform spectrometer (FTS) (Fig. 4f). These two spectra are also close. The Pearson correlation coefficient between measured and retrieved spectrum graphs is 0.91. Small discrepancies are due mainly to noise in the FTS at delays for which the pulse intensity was clearly zero and so yielded spurious additional spectral fine structure. The linear slope in the spectral phase is due to the slightly off-center time of the pulse peak (See Fig. 4c).

## Discussion

In this discussion, we would like to draw the reader’s attention to the assumptions used in the numerical model, as well as to show the discrepancies between the experiment and the numerical simulation. A model for one polarization of radiation was used in the calculations, which in the general case is incorrect when considering fibers in which radiation does not maintain a polarization state. For a more accurate description of the radiation propagation process, it is necessary to solve coupled equations for two orthogonal polarization states^28,29^ that require taking into account the birefringence of fibers, which needs to be measured. Also, the propagation process is highly dependent on the radiation polarization settings, which adds a lot of variabilities in numerical simulations and in performing experiments. Various fluctuations of the environment, vibration, humidity, temperature lead to a change in the birefringence of the fibers and, as a consequence, can change the generation mode of both the laser and the dynamics of pulse propagation in the amplifier. The study of this system for complete pulse polarization both experimentally and theoretically is beyond the scope of this study. Also, the Raman response of germanosilicate fibers is taken the same as for silica fibers, which also introduces errors into the model. For a more accurate description of the amplification in the active fiber, it is necessary to solve the balance equations.

Despite our model assumptions, the numerical results are generally in good agreement with the experiment. The simulation and experiment show a significant broadening of the pulse spectrum at the output of the active fiber with a simultaneous increase in the pulse duration. Thus, in the experiment at the output of the active fiber at a power output of 955 mW the spectral width was 92 nm, and the pulse autocorrelation duration was 8.2 ps. As a result of numerical simulation at a power output of 840 mW, the spectral width was 75 nm, and the pulse duration was 10.4 ps. As a result of pulse compression in the experiment in LMA fiber, the pulse duration was 71 fs and the estimated peak power of 220 kW. As a result of simulation, the pulse at the compression point had a duration of 53.8 fs and a peak power of 236 kW. The compression length differs from the calculated one by 30% (3.12 m in the simulation versus 2.37 m in the experiment).

**Figure 5:**
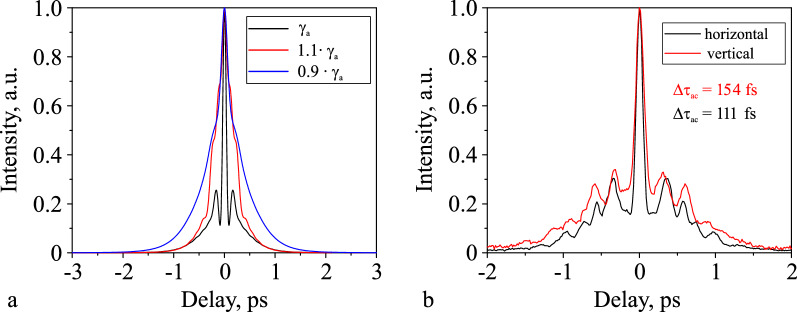
(**a**) Calculated autocorrelation functions of the pulse at the output of the amplifier with Hi-Ge fiber at the LMA fiber length of 3.14 m and the output power of 576 mW. The different color shows the graphs with the nonlinearity coefficient of the active fiber differing by $\pm 10~\%$, which we attribute to the change in the polarization of the radiation before the active fiber. (**b**) Measured autocorrelation functions for two orthogonal polarizations of radiation in the case of a short pulse at polarization controller settings different from the case presented in the main part of the article.

And last we make a few comments as to why the numerical simulation in our case showed a good correspondence with the experiment. Birefringence of fibers and random polarization of radiation lead to two main effects. The first one is the rotation of the polarization ellipse, and its effect leads to different polarizations of pulses in the group, this effect is not considered in the paper, as it requires one to solve the coupled Nonlinear Schrodinger Equation (NLSE). The second effect is the change in the accumulated nonlinear phase shift by the pulse due to a difference of the nonlinear refractive index for different polarizations. Fig. 5a shows the autocorrelation functions at the output of the amplifier with Hi-Ge fiber at a compressor length of 3.1 m and an output power of 576 mW in the case where the active fiber nonlinear coefficient is equal to that calculated (see “Methods” section), and when it is greater or less than the calculated value by 10 percent. As is known, a change in polarization of radiation leads to a change in the nonlinear coefficient^23^. The durations of autocorrelations change significantly with the nonlinear coefficient changes. Thus, we associate the good agreement between the experiment and the model with the nonlinear coefficient variations with polarization, which is selected by adjusting the polarization controllers at which the shortest pulse is formed at the amplifier output. The change in the calculated pulse autocorrelation functions correlates well with the experimental results (Fig. 3d) because a small change in the nonlinear coefficient of the active fiber strongly affects the pulse compression process. There is no certainty if the NLSE can allow good agreement for another system, and for the general case of propagation in non-PM fibers, it is more precise to solve the coupled nonlinear Schrödinger equation. The NLSE allows us to investigate and track general pulse dynamics even in non-PM fiber. However, it cannot guarantee the perfect agreement with the experiment.

Also, we measured autocorrelations for two orthogonal polarizations of radiation in the case of a long pulse and a short one at polarization controller settings different from the case presented in the main part of the article. For the long pulse, the orthogonal autocorrelations were both long. For a short pulse, the orthogonal autocorrelations were similar in duration (differing by 30 percent) and with a slightly different pedestal (Fig. 5b). At the same time, if we implement this amplifier with similar fibers with maintaining polarization, the description of the process without taking into account birefringence will already be more accurate.

## Conclusion

We demonstrated a system for amplification and compression of ultrashort pulses based on thulium-doped normal-dispersion germanosilicate fiber and the LMA silica-fiber compressor. The developed source generates a main pulse with small sub-pulses and has the following characteristics: the main pulse duration of 71 fs, the central wavelength of $1.9~\upmu \hbox {m}$, the repetition rate of 23.8 MHz, the energy per pulse period of 25 nJ, the average power of 600 mW, and the maximum estimated peak power of 220 kW. The dynamics of pulse propagation in the amplifier is analyzed by numerical simulations. As a result of the simulation, it was shown that the use of a stretcher before the active fiber allows increasing the peak power at the compression point and also reducing the energy contained in small subpulses. In addition, the achieved pulse formation was studied using a home-made FROG to determine a correspondence between temporal and spectral characteristics of the pulse. The developed system generates radiation with random polarization and the pulse formation and amplification strongly depend on the PCs settings. The resulting source is suitable for broadband coherent mid-IR supercontinuum generation in different nonlinear media^4,5^. Like all ultrafast fiber systems with non-polarization maintaining fibers, this system is highly sensitive to external influences (temperature, vibration, pressure, etc.). The further optimization of the developed system can be conducted through the employment of polarization-maintaining fibers.

## Methods

The generalized nonlinear Schrödinger equation in the frequency domain used to describe the ultrashort pulse propagation in fibers in the following form^30^:

1 $$\begin{aligned} \begin{aligned} \frac{\partial \tilde{A}'}{\partial z} = i\frac{\gamma \omega }{\omega _0}{\mathrm {exp}}(-{\hat{L}}(\omega )z){\mathscr {F}} \left\{ A(z,t)\int \limits _{-\infty }^{\infty } R(T')|A(z,T-T')|^2dT'\right\} , \end{aligned} \end{aligned}$$

where $\tilde{A}'=\tilde{A}(z,\omega ){\mathrm {exp}}(-{\hat{L}}(\omega )z)$, ${\hat{L}}(\omega )$ is the linear operator, given by ${\hat{L}}(\omega )=i(\beta (\omega )-\beta (\omega _0)-\beta _1(\omega _0)[\omega -\omega _0])-\alpha (\omega )/2$, $\alpha (\omega )$ is the frequency dependent losses or gain, $\beta (\omega )$ is the propagation constant, $\beta _1(\omega _0)$ is the first derivative of the propagation constant, $\omega _0$ is the central angular frequency, $\tilde{A}(z,\omega )$ is the Fourier transform of the normalised amplitude *A*(*z*, *T*) that $|A(z,T)|^2$ gives the instantaneous power in watts, $\gamma =n_2(\omega _0)\omega _0/cA_{\mathrm {eff}}(\omega _0)$ is the nonlinear coefficient, $n_2$ is a nonlinear refractive index, *c* is the speed of light in vacuum, $A_{\mathrm {eff}}(\omega _0)$ is effective mode area, $\omega$ is the angular frequency, $R(T')$ is the Raman response function, *z* is the distance in the waveguide, $T=t-\beta _1z$ is the time in a co-moving frame at the envelope group velocity $\beta _1^{-1}$. Raman response function are defined as^30–32^:

2 $$\begin{aligned} \begin{aligned} R(t)=(1-f_R)\delta (t)+f_Rh_R(t)=(1-f_R)\delta (t)+f_R\frac{\tau _1^2 +\tau _2^2}{\tau _1\tau _2^2}{\mathrm {exp}}(-t/\tau _2){\mathrm {sin}}(t/\tau _1)\Theta (t), \end{aligned} \end{aligned}$$

where $f_R$ represents the fractional contribution of the delayed Raman response to nonlinear polarization, $\Theta (t)$ is the Heaviside step function, $\delta (t)$ is the Dirac delta function, $\tau _1$ is the period of vibrations, $\tau _2$ is the dumping time of vibrations. We use $\tau _1=12.2~\hbox {fs}$, $\tau _1=32.2~\hbox {fs}$, $f_R=0.18$^31^. Differential equation (1) solved by the fourth order Runge-Kutta method using a modified code in Matlab written by J.C. Travers, M.H. Frosz and J.M. Dudley^30^. The gain model of active fiber was used as in the work^22^. The model uses the dispersion of the fibers shown in Fig. 3b. Nonlinear coefficients and effective mode areas at $1.9~\upmu \hbox {m}$ for the fibers used in this work are presented in the Table 1. To calculate the nonlinear coefficient, the value of the nonlinear refractive index was used in proportion to the concentration of germanium oxide in the core^33^. The effective mode area of the fibers was calculated using the Lumerical software.

**Table 1:** Effective mode areas and nonlinear coefficients at 1.9 $\upmu$ m of fibers used in this work, SMF–SMF-28 fiber, Tm—germanosilicate thulium-doped fiber, Hi-Ge—ermanosilicate fiber, LMA—large-mode-area fiber.

Parameters	SMF	Tm	Hi-Ge	LMA
$A_{\mathrm {eff}}$, $\upmu \hbox {m}^{2}$	85.14	11.96	16.16	311.7
$\gamma$, $10^{-3}$/($\hbox {W}\,\hbox {m}$)	0.97	8.85	6.33	0.26

A pulse obtained from the model described in our previous work was used as the pulse input to the amplifier^22^. The difference from the previous work is in the specified parameters of the fibers, which reduced only the maximum calculated average laser power by 2 times. In the simulation, the number of points in the time domain was 8192 along with the width of the time window of 60 ps. When the number of points was doubled, the peak pulse power and the duration of the main pulse changed by less than 1%. The step along the length of the fiber was 1 mm. With decreasing the step up to 0.1 mm, the peak power and duration of the pulse at the compression point changed by less than 0.0065%. To calculate the temporal and spectral FWHM, the calculated curves were approximated by splines into a grid with a number of points greater by a factor of 100.

## Supplementary Information

Supplementary Figures.
