# Supplementary material for: All-fiber ultrafast amplifier at 1.9 μm based on thulium-doped normal dispersion fiber and LMA fiber compressor
Source: Sci Rep. 2021 Dec 8;11:23693. doi: 10.1038/s41598-021-02934-4 (PMC8655054; doi:10.1038/s41598-021-02934-4)
Supplement: Supplementary file 1 — Supplementary Figures. [file 41598_2021_2934_MOESM1_ESM.pdf]

Supplementary materials for: All-fiber ultrafast amplifier at 1.9 µm based on thulium-doped normal dispersion fiber and LMA fiber compressor

Vasilii Voropaev[1], Daniil Batov[1], Andrey Voronets[1], Dmitrii Vlasov[1], Rana Jafari[2], Aleksandr Donodin[3], Mikhail Tarabrin[1,4], Rick Trebino[2], and Vladimir Lazarev[1,*]

[1]Science and Education Center for Photonics and IR-Technology, Bauman Moscow State Technical University, Moscow, 105005, Russia

[2]Georgia Institute of Technology, GA 30332, Atlanta, Georgia, United States

[3]Aston Institute of Photonic Technologies, Aston University, Birmingham, B4 7ET, United Kingdom

[4]Frequency standards Laboratory, P. N. Lebedev Physical Institute of the Russian Academy of Sciences, Moscow, 119991, Russia

[*]Corresponding author: vladimir.al.lazarev@gmail.com

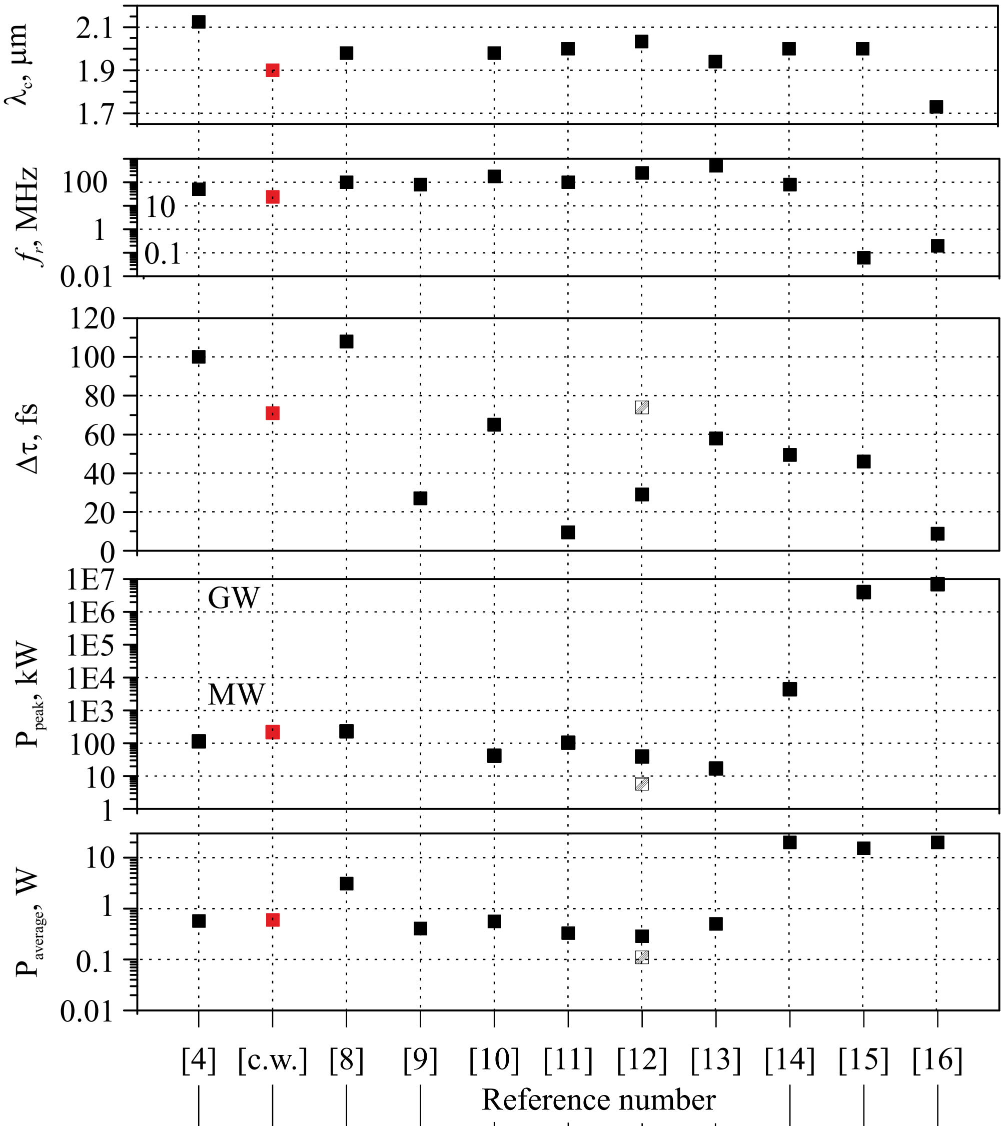

	[4]	[c.w.]	[8]	[9]	[10]	[11]	[12]	[13]	[14]	[15]	[16]
All-fiber	+	+	-	+	+	+	-	-	-	-	-
PM	?	-	-	-	-	+	-	-	?	?	?
LMA	?	+	+	+	-	-	-	-	-	-	-
VLMA	-	-	-	-	-	-	-	-	+	+	+
CPA	+	+	+	?	+	+	-	-	+	+	+
NC	-	-	-	+	+	+	+ -	-	-	+	+
NDA	-	+	-	-	-	-	-	-	-	-	-
Only laser	-	-	-	-	-	-	+	+	-	-	-

Figure 1. Comparison of the characteristics and design features of ultrafast fiber sources based on thulium-doped fibers with a pulse duration less than 150 fs and peak power over 10 kW, which were discussed in the main part of the article. C.W.: current work. Graphs from top to bottom: central wavelength, pulse repetition rate, pulse duration, peak power, average power. All-fiber: a design with no bulk optical components without fiber connectors. PM: schematics with all polarization-maintaining components. LMA: large mode area fibers. VLMA: very large mode area fibers. CPA: chirped pulse amplification. NC: nonlinear pulse compression. NDA: normal dispersion active fibers. Only laser: the works without using amplifiers.

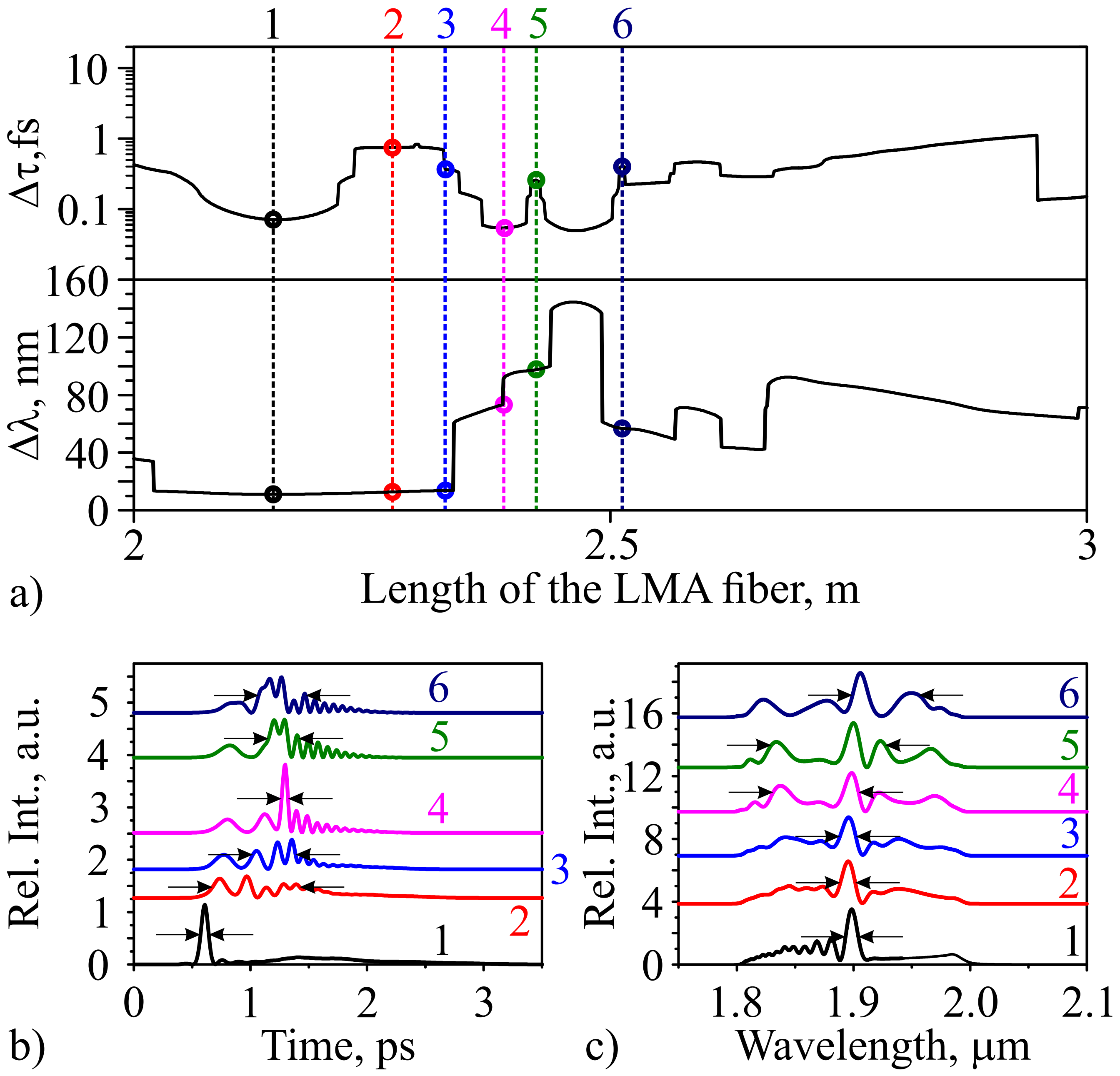

Figure 2. Explanation of the fast oscillations of the pulse group duration and spectrum width near the compression point in the amplifier schematic without Hi-Ge fiber at an average output power of 576 mW. Figure a shows the dependences of the pulse group duration and spectrum width near the compression point with marked key points for which the spectra (c) and temporal intensity profiles (b) are shown below.

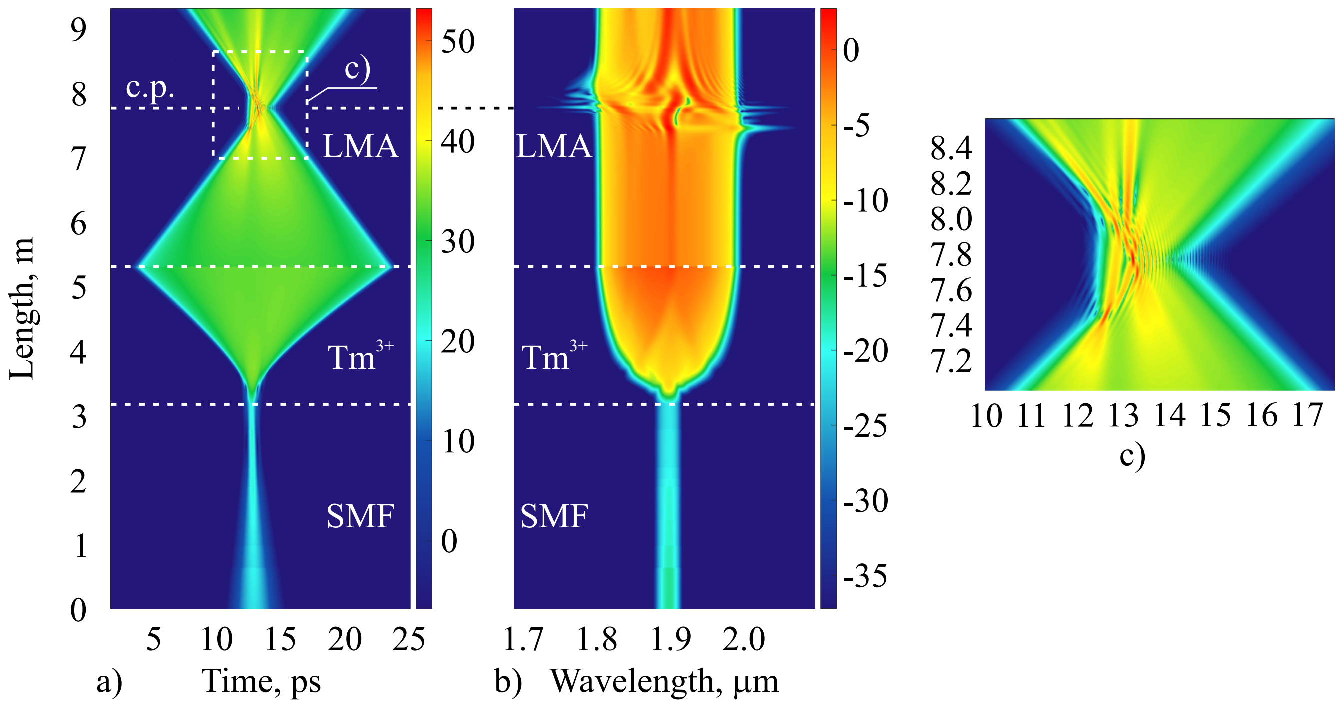

Figure 3. Evolution of the temporal power profile (a) and spectrum (b) of radiation during propagation in the amplifier without Hi-Ge fiber at an average output power of 576 mW. Figure (c) shows an enlarged compression point (c.p.), the axes are the same as in figure (a). The power in the graph (a) is given in dBW. The relative spectral power in the graph (c) is given in dB.

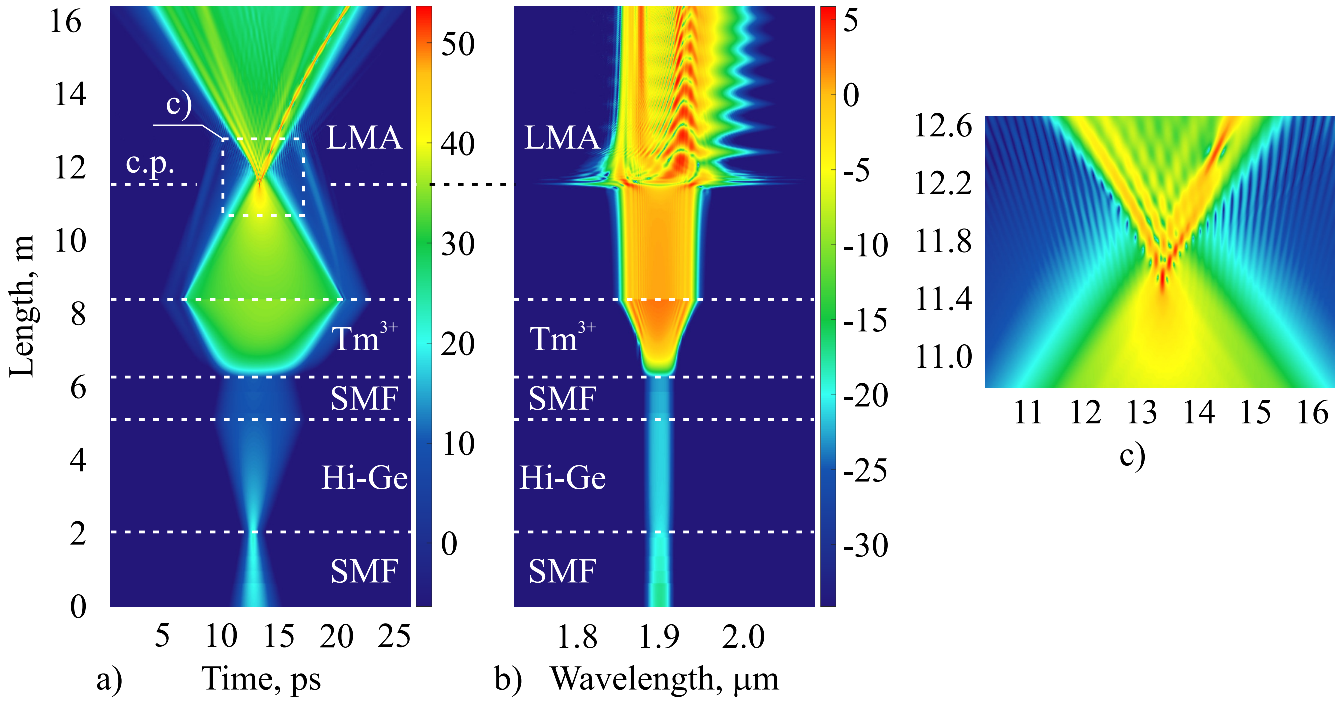

Figure 4. Evolution of the temporal power profile (a) and spectrum (b) of radiation during propagation in the amplifier with Hi-Ge fiber at an average output power of 576 mW. Figure (c) shows an enlarged compression point (c.p.), the axes are the same as in figure (a). The power in the graph (a) is given in dBW. The relative spectral power in the graph (c) is given in dB.
